# Education Loans and Cardiovascular Disease Risk Among Older Adults

**DOI:** 10.1093/geroni/igaf122.110

**Published:** 2025-12-31

**Authors:** Francisco Rios Casas, Christy Erving, Mateo Farina

**Affiliations:** The University of Texas at Austin, Austin, Texas, United States; The University of Texas at Austin, Austin, Texas, United States; University of Texas at Austin, Austin, Texas, United States

## Abstract

Higher levels of educational attainment have been linked to substantially lower cardiovascular risk in later life – a main cause of disability and death in older adulthood. However, due to historic increases in educational debt, the benefits of education for cardiovascular health may be more limited for those that take on student loans. This study uses data from the 2022 wave of the Health and Retirement Study (HRS) to examine the association between outstanding educational debt and health among a population-based sample of US adults between 50 and 80 years of age (N = 10,857). We used logistic regression to examine the association between outstanding debt and four risk factors of cardiovascular disease: diabetes, high blood pressure, obesity, and smoking. Older adults with education debt owed on average $40,000. Moreover, results showed that those with debt were more likely to have diabetes, high blood pressure, and obesity but not more likely to be smokers. This study suggests that outstanding educational loans are increasing in prevalence among recent cohorts of older adults and will be a growing public health concern in the coming decades as more adults continue to take on debt and/or carry it into older adulthood. Additional research is needed to understand the health profiles of older adults that will increasingly reach older adulthood with this type of debt.

